# Glutamate signaling in bone

**DOI:** 10.3389/fendo.2012.00097

**Published:** 2012-08-06

**Authors:** Karen S. Brakspear, Deborah J. Mason

**Affiliations:** ^1^Department of Physiology and Pharmacology, Bristol University,Bristol, UK; ^2^School of Biosciences, Cardiff University,Cardiff, UK

**Keywords:** glutamate, bone, osteoblast, EAAT

## Abstract

Mechanical loading plays a key role in the physiology of bone, allowing bone to functionally adapt to its environment, however characterization of the signaling events linking load to bone formation is incomplete. A screen for genes associated with mechanical load-induced bone formation identified the glutamate transporter GLAST, implicating the excitatory amino acid, glutamate, in the mechanoresponse. When an osteogenic load (10 N, 10 Hz) was externally applied to the rat ulna, GLAST (EAAT1) mRNA, was significantly down-regulated in osteocytes in the loaded limb. Functional components from each stage of the glutamate signaling pathway have since been identified within bone, including proteins necessary for calcium-mediated glutamate exocytosis, receptors, transporters, and signal propagation. Activation of ionotropic glutamate receptors has been shown to regulate the phenotype of osteoblasts and osteoclasts *in vitro* and bone mass *in vivo*. Furthermore, glutamatergic nerves have been identified in the vicinity of bone cells expressing glutamate receptors *in vivo*. However, it is not yet known how a glutamate signaling event is initiated in bone or its physiological significance. This review will examine the role of the glutamate signaling pathway in bone, with emphasis on the functions of glutamate transporters in osteoblasts.

## THE GLUTAMATE SIGNALING PATHWAY

Glutamate is best known for its role as an excitatory signaling molecule in the central nervous system (CNS) where it is involved in learning and memory. Glutamate release from neurons into the synaptic cleft is triggered by Ca^2^^+^ influx through voltage-sensitive calcium channels (VSCC), where it then acts on a variety of receptors at the post-synaptic membrane, resulting in an influx of Ca^2^^+^ into the post-synaptic neuron and propagation of the depolarizing signal. Glutamate receptors can be categorized into ionotropic and metabotropic (iGluR and mGluR respectively). Ionotropic GluRs can be further classified by sequence homology and agonist preference as DL-α-amino-3-hydroxy-5-methylisoxazole-4-propionate (AMPA), kainate (KA), and *N*-methyl-D-aspartate (NMDA), which are associated with ion channels permeable to specific cations (Wisden and Seeburg, 1993; Hollmann and Heinemann, 1994). Metabotropic GluRs are G-protein coupled and are categorized into three functional groups based on their sensitivity to exogenous agonists and intracellular signaling mechanisms; group I (mGluR1 and mGluR5), group II (mGluR2 and mGluR3), and group III (mGluR4, mGluR6, mGluR7, and mGluR8; Masu et al., 1991; Tanabe et al., 1992; Wisden and Seeburg, 1993). High-affinity glutamate transporters (excitatory amino acid transporters; EAATs) at the pre-synaptic and post-synaptic membrane and neighboring glial cells terminate the signaling event by removing glutamate from the synaptic cleft.

There is strong evidence to suggest that glutamate signaling pathways are functional in several sites of the body besides the CNS and bone (reviewed in Skerry and Genever, 2001; Hinoi et al., 2004). Glutamate signaling mechanisms are able to detect very fast stimulatory signals and self-modify, making them well-suited for responding to mechanical signaling in bone (Skerry, 2002; Turner et al., 2002; Spencer and Genever, 2003; Bowe and Skerry, 2005).

## GLUTAMATE RELEASE

Osteoblasts express the functional components required for regulated neuronal glutamate release including molecules involved in synaptic vesicle packaging, targeting, and fusion (Bhangu et al., 2001; Bhangu, 2003). Osteoblasts spontaneously release glutamate *in vitro* (Genever and Skerry, 2001; Hinoi et al., 2002a) and glutamate release by rat calvarial osteoblasts is increased following depolarization with 50 mM KCl or activation of iGluRs with AMPA (Hinoi et al., 2002a). The initiating stimulus for glutamate release in osteoblasts remains unclear, though Mason (2004) proposed that mechanical load may open stretch-sensitive calcium channels in osteocytes to trigger glutamate release by osteocytes and activate osteoblast receptors. Interestingly, the intracellular glutamate concentration is regulated during osteoblast differentiation through the action of glutamine synthetase (GS), which converts glutamate to glutamine (Olkku and Mahonen, 2008). During osteogenic differentiation of rat mesenchymal stem cells (MSCs), GS activity declines rapidly at the onset of mineralization increasing intracellular glutamate concentrations (Olkku and Mahonen, 2008; Zheng and Quirion, 2009).

Mature osteoclasts, but not pre-osteoclasts, release glutamate and bone degradation products from transcytotic vesicles following depolarization with 50 mM KCl and this release is dependent on extracellular Ca^2^^+^ (Morimoto et al., 2006).

## GLUTAMATE RECEPTOR EXPRESSION AND FUNCTION

### IONOTROPIC RECEPTORS

Various glutamate receptor subunits are expressed and functional in bone cells (**Table 1**). Both glutamate and NMDA elicit significant increases in membrane currents in MG-63 and SaOS-2 osteoblast-like cells (Laketic-Ljubojevic et al., 1999) and in rabbit primary osteoclasts (Espinosa et al., 1999; Peet et al., 1999), which can be inhibited by the NMDA receptor antagonist MK-801. NMDA receptors are also expressed and functional in primary cultures of rat osteoblasts, with electrophysiological and pharmacological characteristics similar to neuronal NMDA receptors (Gu et al., 2002).

**Table 1:** Reported transcript and protein expression of glutamatergic signaling components in bone cells.

	Osteoblast	Osteoclast	Osteocyte
**NMDA**			
mRNA^1–7^	NMDAR1*,2A,	NMDAR1*,2A,	
	2B,2C,2D	2B,2C,2D	
Protein^1,4,5,6,8-10^	NMDAR1*,2D*	NMDAR1*,2A*,	NMDAR1*
		2B*,2C*,2D*	
**AMPA**			
mRNA^11^	GluR3, 4		
Protein^8,9^	GluR1,2	GluR1,2*,3*,4*	GluR1*,2*
**Kainate**			
mRNA^12^	KA1,2		
Protein^9^		GluR5*,6*,7*	
**mGluR**			
mRNA^2,3,13^	mGluR1b,4,8	mGluR3,4,5,8	
Protein^13^		mGluR8	
**EAAT**			
mRNA^14–21^	EAAT1*,2,3	EAAT2,4	EAAT1*,2
Protein^14,16,19–21^	EAAT1*	EAAT4	EAAT1*

1 Patton et al. (1998);

2 Gu and Publicover (2000);

3 Hinoi et al. (2001);

4 Itzstein et al. (2000);

5 Itzstein et al. (2001);

6 Hinoi et al. (2003);

7 Merle et al. (2003);

8 Chenu et al. (1998);

9 Szczesniak et al. (2005);

10 Ho et al. (2005);

11 Hinoi et al. (2002b);

12 Hinoi et al. (2002a);

13 Morimoto et al. (2006);

14 Mason et al. (1997);

15 Huggett et al. (2000);

16 Kalariti et al. (2004);

17 Takarada et al. (2004);

18 Hinoi et al. (2004);

19 Kalariti et al. (2007);

20 Hinoi et al. (2007);

21 Takarada and Yoneda (2008).

* Denotes in vivo expression.

### METABOTROPIC RECEPTORS

Transcripts have been detected for mGluR1b in rat femoral osteoblasts (Gu and Publicover, 2000) and for mGluR4 and mGluR8 in rat calvarial osteoblasts (Hinoi et al., 2001). Upon exposure of rat femoral osteoblasts to 1*S*,3*R*-ACPD, an mGluR agonist, elevated levels of intracellular Ca^2+^ were observed, indicating functional group I mGluR expression (Gu and Publicover, 2000). The initial phase of this response was not dependent on extracellular Ca^2+^ levels, reflecting mobilization of Ca^2+^ from intracellular stores (Gu and Publicover, 2000). In rat calvarial osteoblasts, a group III mGluR agonist significantly inhibited forskolin-induced cAMP accumulation in a manner that could be prevented by co-treatment with a group III mGluR antagonist, indicating functional group III mGluR expression (Hinoi et al., 2001). Expression of mGluR6 has also been detected in rat bone marrow stromal cells (Foreman et al., 2005). In these cells, treatment with glutamate resulted in inhibition of Ca^2+^ influx and subsequent membrane hyperpolarization which was sensitive to the group III mGluR antagonist (s)-MAP4, suggesting that activation of mGluR6 inhibits a Ca^2+^-permeable membrane channel (Foreman et al., 2005).

Interestingly, NMDA currents in rat femoral osteoblasts that were lost upon treatment with glutamate could be restored by blockade of mGluRs, indicating that inhibitory cross talk occurs between mGluRs and NMDA receptors in osteoblasts (Gu and Publicover, 2000).

## GLUTAMATE RECEPTOR REGULATION

Expression of iGluR subunits is regulated by mechanical load in bone (Szczesniak et al., 2005). Long bones of adult rats subjected to cyclic compressive load for four consecutive days displayed a load-induced loss of immunoreactivity to various iGluR subunits in osteoclasts and bone lining cells (Szczesniak et al., 2005). Furthermore, NMDA receptor expression was down-regulated in osteoblasts in association with disuse-induced bone loss in rats (Ho et al., 2005).

## SECONDARY SIGNALING AND PHENOTYPIC EFFECTS

### OSTEOBLASTS

The secondary signaling pathways following glutamate receptor activation of osteoblasts have not been well-characterized, although activation of receptor-associated protein kinases and translocation of the transcription factor activator protein-1 (AP-1) has been demonstrated (Taylor, 2002; Lin et al., 2008; Li et al., 2011). Antagonists to NMDA receptors down-regulate the transcription factor Runx2 and inhibit alkaline phosphatase activity and osteocalcin expression in rat primary osteoblasts (Hinoi et al., 2003; Ho et al., 2005). Consistent with this, more recent studies have shown that the AMPA receptor antagonist 6-cyano-7-nitroquinoxaline-2,3-dione (CNQX) and the NMDA receptor antagonist MK-801 inhibit rat calvarial osteoblast activity and mineralization, whilst the agonists, AMPA and NMDA, up-regulate osteocalcin expression and mineralization of osteoblasts in glutamate-free medium (Lin et al., 2008). *In vivo* evidence also indicates an important role for glutamate signaling in bone formation. Injection of AMPA locally into the tibia of young rats increased bone volume in a manner that was prevented by CNQX (Lin et al., 2008). Furthermore, mice treated with the AMPA receptor antagonist NBQX or the NMDA receptor antagonist AP5 by osmotic minipumps over 8 days, exhibited altered bone structure (Burford et al., 2004). Trabecular thickness was reduced in NBQX-treated mice whereas cortical thickness at midshaft sites was reduced in AP5-treated mice and increased in NBQX-treated mice (Burford et al., 2004). This indicates different roles for NMDA and AMPA receptors in the regulation of trabecular and cortical bone mass (Burford et al., 2004; Skerry, 2008). Finally, osteocalcin promoter-driven knockout of NMDAR1 in mice causes stunted skeletons indicative of a role for glutamate signaling in skeletal development (Skerry, 2008).

### OSTEOCLASTS

Activation of NMDA receptors in osteoclasts influences cellular phenotype *in vitro*. In co-cultures of mouse bone marrow leukocytes and osteoblasts in which osteoclasts differentiate, MK-801 suppressed osteoclast differentiation and reduced resorption pit formation in dentine (Peet et al., 1999). No significant effects of MK-801 upon mature osteoclast activity could be discerned in this study (Peet et al., 1999), although others have reported that MK-801 inhibits mature rabbit osteoclast activity (Chenu et al., 1998; Itzstein et al., 2000) and promotes apoptosis, via decreased NO production (Mentaverri et al., 2003). Glutamate and NMDA receptor agonists induced nuclear translocation of nuclear factor-kappaB (NF-κB) in osteoclast precursor cell lines, and this was inhibited by MK-801 (Merle et al., 2003) indicating that glutamate-mediated activation of the NF-κB pathway is involved in osteoclastogenesis.

## GLUTAMATE TRANSPORTERS

### HIGH-AFFINITY TRANSPORTERS

In the CNS, extracellular glutamate concentrations are tightly controlled by high-affinity, sodium-dependent glutamate transporters. These transporters (EAATs) are classified into five subtypes (EAAT1/GLAST, EAAT2/GLT-1, EAAT3/EAAC1, EAAT4, and EAAT5; Kanai and Hediger, 1992; Pines et al., 1992; Storck et al., 1992; Tanaka, 1993; Arriza et al., 1994, 1997; Fairman et al., 1995). Several splice variants of EAATs 1–3 have been reported, which have altered function and expression profiles (Lin et al., 1998; Nagai et al., 1998; Matsumoto et al., 1999; Meyer et al., 1999; Huggett et al., 2000; Vallejo-Illarramendi et al., 2005). Each EAAT subtype displays heterologous spatial and cellular expression profiles (Danbolt, 2001) indicating a complex and finely tuned control over extracellular glutamate levels.

Excitatory amino acid transporters transport glutamate against its concentration gradient. Three sodium ions and one proton are co-transported with glutamate and one potassium ion is counter-transported leading to a net positive charge moving into the cell (Zerangue and Kavanaugh, 1996). EAATs also function as ion channels whereby sodium-dependent glutamate binding initiates an uncoupled anion conductance (Seal and Amara, 1999; Slotboom et al., 2001) that is physiologically measurable (Billups et al., 1996; Eliasof and Jahr, 1996). The role of the anion conductance is unclear, though it has been suggested that it may compensate for the membrane potential changes due to electrogenic glutamate uptake (Billups et al., 1996; Eliasof and Jahr, 1996). The chloride conductance may also modulate glutamate receptor activity or operate like a receptor, activating intracellular signaling cascades in response to glutamate binding (Danbolt, 2001; Mason and Huggett, 2002). In addition to transport and ion channel activities, protein–protein interactions with both N- and C-terminal domains of the EAATs suggest a potential receptor-like function for the EAATs. Interactions with the actin cytoskeleton and the mitogen-activated protein kinase (MAPK) cascade have been reported (Abe and Saito, 2001; Marie et al., 2002; Sullivan et al., 2007).

### EAAT EXPRESSION AND FUNCTION IN BONE

GLAST and GLT-1 are expressed in osteoblasts and osteocytes *in vivo* (Mason et al., 1997) and EAAT3 has been detected in rat primary osteoblasts *in vitro* (Takarada et al., 2004; **Table 1**). In contrast, EAATs 2 and 4 appear to be the predominant EAATs in osteoclasts (Hinoi et al., 2007; Takarada and Yoneda, 2008). GLAST-1a, a splice variant lacking domains important for anion conductance is also expressed in bone *in vivo* (Huggett et al., 2000).

Despite EAATs being the first component of glutamatergic signaling to be identified in bone, the majority of glutamate signaling research within bone has focused on the activity of the glutamate receptors. It has been hypothesized that the EAATs might play a direct role in regulating the phenotype of bone cells via their various activities; glutamate uptake, glutamate release, glutamate-gated ion channel, or activation of intracellular signaling pathways (Mason, 2004). This has been supported by studies reporting that the EAAT inhibitor *t*-PDC prevents bone formation of calvarial osteoblasts *in vitro* (Taylor, 2002) and our data showing that pharmacological EAAT inhibition can influence the bone-forming phenotype of osteoblast-like cells *in vitro* (Brakspear et al., 2009).

*In vivo* evidence that GLAST is expressed in bone where it is mechanically regulated in osteocytes and osteoblasts (Mason et al., 1997) indicates that the transport system is physiologically relevant (Mason et al., 1997). Although, knockout of GLAST has been reported to have no affect on bone length (Gray et al., 2001), the role of GLAST in bone remodeling and responses to load remains unknown (Chenu et al., 2001; Skerry et al., 2001).

We have observed that EAAT subtypes are differentially expressed across MG-63, SaOS-2, and human primary osteoblasts – EAAT1 mRNA is expressed at high levels compared to EAAT3 while the expression of EAAT2 is low and this varies with each cell type (Brakspear et al., 2009). This complicates interpretation of the effects of EAAT inhibition since uptake kinetics, Cl^−^ conductance, post-translational regulation and protein–protein interactions (reviewed in Danbolt, 2001) vary across the EAAT subtypes. The localization of different EAAT subtypes within the cell is tightly regulated (reviewed in Danbolt, 2001; Amara and Fontana, 2002). EAAT localization adjacent to specific glutamate receptor subtypes will modulate the glutamate available for receptor activation, influencing intracellular signaling events. For example glutamate uptake through EAAT4 limits mGluR activation in Purkinje neurons in the cerebellum (Wadiche and Jahr, 2005) and GLAST restricts the activation of mGluRs in hippocampal neurons (Huang et al., 2004). The intracellular localization of EAATs are differentially regulated by extracellular glutamate concentrations in osteoblasts (Huggett et al., 2002), consistent with the notion that EAATs may exhibit similar mechanisms of regulating glutamate receptor activation in bone cells.

## CYSTINE/GLUTAMATE ANTIPORTERS

Cystine/glutamate antiporters are sodium-independent, chloride-dependent high-affinity glutamate transporters (Bannai, 1986; Sato et al., 1999). The transporter is a heterodimer of the CD38 heavy chain (also called 4F2hc) and the $X_{C}^{-}$ (referred to as xCT) light chain. Cystine (the dimeric form of cysteine) is necessary for the generation of the tripeptide antioxidant glutathione (γ-Glu-Cys-Gly; GSH; reviewed in Cooper and Kristal, 1997; Dringen, 2000). The cystine/glutamate antiporter is expressed and functionally required for the differentiation of pre-osteoblasts, pre-osteoclastic RAW264.7 cells, and primary osteoclasts from bone marrow precursor cells (Hinoi et al., 2007; Takarada-Iemata et al., 2010). Differentiation was inhibited by high glutamate concentrations (over 500 μM) in a manner that was sensitive to inhibitors of the antiporter. High concentrations of extracellular glutamate are likely to result in cystine being released from the cell, thus reducing intracellular cystine available to generate GSH. The cystine/glutamate antiporter is also expressed in undifferentiated MC3T3-E1 osteoblast-like cells where it suppresses proliferation, without inducing cell death, following treatment with exogenous glutamate in association with decreased levels of intracellular GSH (Uno et al., 2007). Interestingly, recent findings have indicated that the activity of the antiporter down-regulates Runx2 expression and alkaline phosphatase activity in MC3T3-E1 cells and mouse calvarial osteoblasts under differentiating conditions (Uno et al., 2011) suggesting that the role of the antiporter in bone cells may be maturation-stage specific. The intracellular concentration of glutamate increases at the onset of mineralization (Olkku and Mahonen, 2008; Zheng and Quirion, 2009), which may explain the stage-specific inhibitory activity of the antiporter.

## VESICULAR GLUTAMATE TRANSPORTERS

Vesicular glutamate transporters (VGLUTs) package glutamate into vesicles for exocytic release. Expression of VGLUT1, but not VGLUT2, has been detected in rat calvarial osteoblasts (Hinoi et al., 2002a) and glutamate release from these cells was Ca^2+^-dependent and sensitive to AMPA GluR antagonists (Hinoi et al., 2002a). VGLUT1 is also expressed by mature osteoclasts and is thought to accumulate glutamate into transcytotic vesicles for release, with bone degradation products, upon stimulation with 50 mM KCl or ATP in a Ca^2+^-dependent manner (Morimoto et al., 2006). Glutamate released from osteoclasts by transcytosis may be autoregulatory, since agonists to osteoclastic mGluR8 inhibits secretion of glutamate and bone degradation products whereas mGluR8 antagonists stimulate bone resorption (Morimoto et al., 2006).

## PATHOLOGICAL IMPLICATIONS OF GLUTAMATE SIGNALING IN OSTEOBLASTS

**FIGURE 1:**
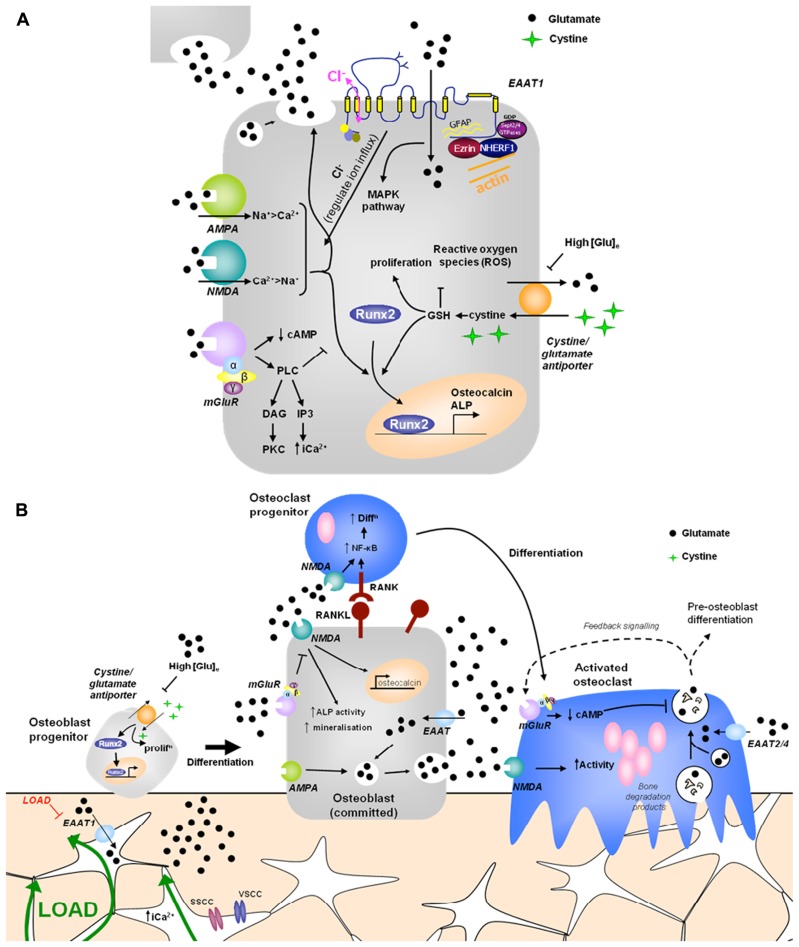
**Hypothetical model of glutamate signaling in (A) osteoblasts and (B) bone. (A)** Osteoblasts release glutamate to activate glutamate receptors in an autocrine and paracrine manner and express functional ionotropic and metabotropic glutamate receptors. iGluR activation leads to glutamate release and increased Runx2 activity, regulating osteocalcin expression, ALP activity and mineralization (Hinoi et al., 2002c, 2003; Ho et al., 2005). mGluR activation inhibits NMDA receptor signals in osteoblasts via PLC activated pathways (Gu and Publicover, 2000). High extracellular [glutamate] inhibits the cystine/glutamate antiporter, suppressing proliferation and reducing Runx2 activity due to depletion of GSH (Uno et al., 2007; Takarada-Iemata et al., 2010). EAATs transport glutamate into osteoblasts, modulating localized receptor responses, but also activating a chloride flux, which may function as a voltage clamp, act directly as a receptor, or modulate GluR activation by regulating ion influx (Danbolt, 2001; Huggett et al., 2002). Various proteins interact with the intracellular domains of EAAT1 and glutamate transport through EAATs activates MAPK (Abe and Saito, 2001). **(B)** Opening of stretch- and voltage-sensitive calcium channels (SSCC, VSCC) in osteocytes in response to mechanical load increases intracellular [Ca^2+^] to induce glutamate release into junctions with neighboring osteocytes (Mason, 2004). Down-regulation of EAAT1 in mechanically loaded osteocytes (Mason et al., 1997) would increase extracellular [glutamate] which could regulate osteoblast differentiation and activity as described above. Osteoclasts are also likely to be regulated by released glutamate. Osteoclasts express EAATs 2 and 4 (Hinoi et al., 2007; Takarada and Yoneda, 2008) and NMDA receptor activation promotes NF-κB stimulated osteoclast differentiation (Peet et al., 1999; Merle et al., 2003) and increases mature osteoclast activity (Chenu et al., 1998; Itzstein et al., 2000; Mentaverri et al., 2003). Mature osteoclasts release glutamate in conjunction with bone degradation products, which can act on autoregulatory mGluRs, preventing further glutamate release (Morimoto et al., 2006). Therefore, glutamate signals may contribute to mechanical cues and coupling of bone remodeling. PLC, phospholipase C; DAG, diacylglycerol; cAMP, cyclic adenosine monophosphate; PKC, protein kinase C; IP3, inositol triphosphate; ALP, alkaline phosphatase; ROS, reactive oxygen species; MAPK, mitogen-activated protein kinase; EAAT, excitatory amino acid transporter; GSH, glutathione.

Glutamatergic signaling was first discovered in bone in a screen to identify genes associated with mechanically induced bone formation (Mason et al., 1997). Since then there has been substantial evidence that glutamate signaling can modulate osteoblast differentiation and activity, via two opposing mechanisms (**Figure 1A**).

Proliferation and osteoblast differentiation of MC3T3-E1 and mesenchymal C3H10T1/2 stem cells infected with Runx2 adenovirus were prevented by high concentrations of glutamate which inhibit the cystine/glutamate antiporter and reduce GSH levels (Uno et al., 2007; Takarada-Iemata et al., 2010), whereas glutamate activation of the NMDA receptor increases Runx2 and ALP activity of rat calvarial osteoblasts if added after the proliferative stage of maturation (Hinoi et al., 2003). These data suggest that high concentrations of glutamate inhibit pre-osteoblast differentiation in association with GSH depletion, but that activation of specific glutamate receptors increases differentiation and bone-forming activity of committed osteoblasts. Indeed, overexpression of the cystine/glutamate antiporter xCT subunit in differentiating MC3T3-E1 cells negatively regulated Runx2 expression in these cells, indicating an altered role for the antiporter, and thus the effects of extracellular glutamate on this antiporter, in committed osteoblasts (Uno et al., 2011). These different mechanisms may reflect changes in the expression and activity of glutamate signaling components during maturation of osteoblasts, or the influence of different extracellular glutamate concentrations, i.e., pathophysiological concentrations inhibit the cystine/glutamate antiporter whereas physiological concentrations activate glutamate receptors. Intriguingly, synovial fluid glutamate concentrations are greatly increased in osteoarthritis and rheumatoid arthritis (RA; McNearney et al., 2004) where major disruption in bone remodeling occurs. Elucidation of the influence of extracellular glutamate concentrations, maturation-stage, and the diversity of subunits and splice variants that contribute to each receptor/transporter is essential for progress toward therapeutic targeting of glutamatergic in the treatment of bone and joint disorders.

## INTERACTIONS WITH OTHER SIGNALS

Evidence from the CNS suggests interplay may exist between glutamate and other signaling pathways that are important in bone such as insulin-like growth factor (IGF), adenosine, and calcium (Gama et al., 2001; Ferre et al., 2002; Garcia-Galloway et al., 2003; Domenici et al., 2004; Ciruela et al., 2006; Zheng and Quirion, 2009). IGF has been implicated in mechanical load-induced osteogenesis, while adenosine and calcium receptors can influence bone formation (Dvorak and Riccardi, 2004; Dvorak et al., 2004; Evans et al., 2006). Activation of PLC by parathyroid hormone (PTH) in rat femoral osteoblasts prevented calcium influx through NMDA receptors suggesting interplay between these two pathways in bone (Gu and Publicover, 2000). Furthermore, the canonical Wnt signaling pathway negatively regulates the activity of GS in MG-63 cells, thus increasing the intracellular glutamate concentration (Olkku and Mahonen, 2008).

## SUMMARY

A physiological role for glutamate in the regulation of bone mass has been highlighted by transgenic models lacking components of the signaling pathway which display altered bone structure. Furthermore, glutamate receptor activation regulates osteoblast and osteoclast differentiation and activity, and osteogenic mechanical load regulates expression of GLAST and glutamate receptors in bone *in vivo* (Mason et al., 1997; Ho et al., 2005; Szczesniak et al., 2005). The influence of glutamate in bone depends upon the cell type, the differentiation stage and the extracellular glutamate concentration (**Figure 1B**). As in the CNS, glutamatergic signaling in bone is highly complex involving multiple components with different roles in various cell types.

High glutamate concentrations in synovial fluids of RA patients activate nerves within the joint to cause pain. Since, many joint tissues express functional glutamate transporters and receptors, and release this signal, the role of glutamatergic signaling in coordinating joint loading to symptoms of pain, inflammation and joint destruction is of great interest.

## Conflict of Interest Statement

The authors declare that the research was conducted in the absence of any commercial or financial relationships that could be construed as a potential conflict of interest.
